# Programmed Cell Death Ligand 1-Transfected Mouse Bone Marrow Mesenchymal Stem Cells as Targeted Therapy for Rheumatoid Arthritis

**DOI:** 10.1155/2021/5574282

**Published:** 2021-08-30

**Authors:** Qiong-ying Hu, Yun Yuan, Yu-chen Li, Lu-yao Yang, Xiang-yu Zhou, Da-qian Xiong, Zi-yi Zhao

**Affiliations:** ^1^Department of Laboratory Medicine, Hospital of Chengdu University of Traditional Chinese Medicine, Chengdu, Sichuan 610072, China; ^2^College of Medical Technology, Chengdu University of Traditional Chinese Medicine, Chengdu, Sichuan 610072, China; ^3^Department of Thyroid and Vascular Surgery, The Affiliated Hospital of Southwest Medical University, Luzhou 646000, China; ^4^Central Laboratory, Hospital of Chengdu University of Traditional Chinese Medicine, Chengdu, Sichuan 610072, China; ^5^TCM Regulating Metabolic Diseases Key Laboratory of Sichuan Province, Hospital of Chengdu University of Traditional Chinese Medicine, Chengdu, Sichuan 610072, China

## Abstract

Programmed cell death 1 ligand (PD-L1) and its receptor (PD-1) are key molecules for immunoregulation and immunotherapy. PD-L1 binding PD-1 is an effective way to regulate T or B cell immunity in autoimmune diseases such as rheumatoid arthritis (RA). In our study, we overexpressed PD-L1 by constructing a recombinant of PD-L1-lentiviral vector, which was subsequently used to transfect mouse bone marrow mesenchymal stem cells (MBMMSCs) and significantly suppressed the development of collagen-induced arthritis (CIA) in DBA/1j mice. In addition, PD-L1-transfected MBMMSCs (PD-L1-MBMMSCs) ameliorated joint damage, reduced proinflammatory cytokine expression, and inhibited T and B cell activation. Furthermore, PD-L1-MBMMSCs decreased the number of dendritic cells and increased the numbers of regulatory T cells and regulatory B cells in joints of CIA mice. In conclusion, our results provided a potential therapeutic strategy for RA treatment with PD-L1-MBMMSC-targeted therapy.

## 1. Introduction

Rheumatoid arthritis (RA) is a chronic systemic autoimmune inflammatory disease characterized by synovitis in small- and medium-sized joints and joint damage and destruction that affects 1-2% of adults, and it is estimated that approximately 23.7 million people live with RA worldwide [1]. The current theory is that RA is involved in cross-talk between multiple systems and multiple cell types and results from interactions between activated Th1 and Th17 cells, the secretion of proinflammatory cytokines such as tumor necrosis factor (TNF) or interleukin (IL) by infiltrated macrophages, and B cell production of antibodies [2, 3]. These mediators or factors are potential targets for immunomodulatory or immunotherapy. With advances of immunosuppressants and biological drugs, patients with RA have relatively increased remission rates, although despite remission in 30–40% of patients, joint destruction still occurs in RA patients, and fundamental treatments for RA are still insufficient [4]. In addition to TNF inhibitors and other standard therapies, RA treatment involves targeting pathways, such as B7 family members (programmed cell death 1 ligand (PD-L1)) [5]. Preliminary studies confirmed that in mouse RA models, PD-1 or PD-L1 deficiency exacerbated RA [6, 7]. Another study showed that the expression of PD-L1 on B cells was significantly decreased in untreated RA patients but increased in successfully treated RA patients [8].

PD-L1 is one ligand of its receptor PD-1, and the binding of PD1 and PD-L1 triggers negative signaling, inhibiting T cell activation and cytokine production [9]. As a member of the B7/CD28 family of costimulatory molecules, PD-L1 is a cell surface glycoprotein on T and B cells that suppresses host immune functions [10]. As previously described, because it can suppress T cell activity, PD-L1 has rapidly led to the development of treatments or prognostic markers for cancers [11–13]. It is also believed that normal cells expressed PD-L1 in an inflammatory environment to prevent excessive tissue or organ damage from persistent and spreading inflammation. PD-L1 binding to PD-1 on activated T cells promotes tyrosine phosphorylation in the immunoreceptor tyrosine-based switch motif (ITSM) domain of PD-1, which subsequently causes dephosphorylation of the downstream protein kinases spleen tyrosine kinase (Syk) and phosphatidylinositol 3-kinase (PI3K) and inhibits the activation of downstream AKT and ERK signaling. These factors ultimately suppress the transcription and translation of genes and cytokines required for T cell activation and play negative roles in regulating T cell activity [14]. Based on its immunoregulatory function, the PD-1/PD-L1 pathway appears to be an immune checkpoint to control inflammation in RA.

Mesenchymal stem cells (MSCs) are multipotent cells that can differentiate into different cell types, have low immunogenicity and exhibit remarkable immunomodulatory effects, and have been shown to be potential treatments for various autoimmune disease or gene therapy vectors [15, 16]. MSCs were first discovered in the bone marrow; thus, mouse bone marrow mesenchymal stem cells have been extensively studied in the context of immune cells [17]. A primary study compared the therapeutic effects of different mesenchymal stem cells on rheumatoid arthritis in mice, and the authors found that both bone marrow mesenchymal stem cells and umbilical cord mesenchymal stem cells significantly alleviated RA [18]. Increasingly, recent developments in transfection methods and gene delivery have facilitated the delivery of exogenous DNA or RNA to MSCs to alter gene expression by viral gene delivery systems and gene therapy technologies [19, 20].

In short, both PD-1/PD-L1 pathway and MSCs have the ability to regulate immune reactions. However, the individual mechanisms and their synergistic effects are not clear. Here, we developed a lentiviral PD-L1 overexpression system by transfecting MSCs to induce continuously high protein expression of PD-L1 and enhance PD-L1 and MSC efficacy in immunomodulation and immunotherapy. We hypothesized that PD-L1-transfected MBMMSCs (PD-L1-MBMMSCs) would effectively suppress the development of collagen-induced arthritis in DBA/1j mice. Furthermore, we examined the alterations in RA-related immune cells and the generation of proinflammatory cytokines.

## 2. Materials and Methods

### 2.1. Mice

Male 8-week-old DBA/1j mice were purchased from Beijing Hua Fu Kang Biological Technology Co., Ltd. (Beijing, China) and maintained in a pathogen-free animal care facility. All animal care and experimental procedures were performed according to the regulations of the Animal Care Committee of Chengdu University of Traditional Chinese Medicine.

### 2.2. Cell Lines

MBMMSCs were isolated from DBA/1j mice. Long bones of 4–6 weeks male mice were collected to harvest bone marrow. The hindlimbs of male mice were split by cutting off the knee. And the ends of bone were cut with a sharp pair of scissors. Be careful not to splinter the bones during the cutting process. Then, the bone marrow was washed with 1 ml syringe, and the washing solution was culture medium *α*-MEM. The cells were filtered through a 70 *μ*m strainer and collected with a 50 ml centrifuge tube. Bone marrow cells are diluted in complete culture medium (*α*-MEM, 10% FBS, 100 U/ml penicillin, and 100 *μ*g/ml streptomycin) and cultured at 37°C with 5% CO_2_. To confirm the MSCs, FACS were conducted to detect the feature of MSCs, such as CD105^+^ (MJ7/18, BD Biosciences, USA), CD73^+^ (TY/23, BD Biosciences, USA), CD90^+^ (53-2.1, BD Biosciences, USA), CD34^−^ (RAM34, BD Biosciences, USA), and CD11b^−^ (M1/70, BD Biosciences, USA) in Figure S1. Rat IgG2a *κ* Isotype (R35-95) and Rat IgG2b *κ* Isotype (A95-1) were also bought from BD Biosciences. MBMMSCs were maintained in UltraCULTURE™ medium (Lonza, USA).

### 2.3. Construction of the PD-L1-Lentiviral Vector

To amplify PD-L1 sequence, two pairs of primers were designed and synthesized based on the PD-L1 cDNA sequence (NM-001111283.2). The sequences both contained *Xba*I and *Not*I restriction enzyme site.

### 2.4. Virus Production

The PD-L1 gene was cloned into the lentiviral shuttle plasmid PCDH and 293T cells were cotransfected with the recombinant plasmid and the packaging plasmids PMD2.G and psPAX2 using the standard calcium phosphate precipitation method according to the Calcium Phosphate Cell Transfection Kit (Beyotime Biotechnology, China).

### 2.5. Transduction of MBMMSCs

MBMMSCs (1.0 × 10^5^) were seeded in a 6-well plate and incubated overnight. On the following day, the culture media was removed, and the MBMMSCs were transduced with 1 ml recombinant PD-L1-lentivirus at a multiplicity of infection (MOI) of 10 at 37°C, 5% CO_2_. After 8 hours, the transduction media was replaced with fresh DMEM supplemented with 10% FBS at 37°C and 5% CO_2_. After 24 hours, puromycin (4 *μ*g/ml) was added to each well to screen the transduced MBMMSCs. After 3 days of screening, the growth morphology of the MBMMSCs was observed. The overexpression of PD-L1 was confirmed with anti-PD-L1 (MIH5, BD Biosciences, USA) by flow cytometry.

### 2.6. Animal Experiment

DBA/1j mice were immunized with bovine CII (Chondrex, Seattle, WA, USA) to establish a CIA model. When most of the mice showed features of CIA, the mice were randomly assigned to four groups as follows: (a) PBS (treated with 100 *μ*l PBS), (b) MBMMSCs (treated with 100 *μ*l PBS containing 5 × 10^6^ MBMMSCs), (c) Vector-MBMMSCs (treated with 100 *μ*l PBS containing 5 × 10^6^ MBMMSCs transfected with lentiviral vector), and (d) PD-L1-MBMMSCs (treated with 100 *μ*l PBS containing 5 × 10^6^ MBMMSCs transfected with PD-L1 lentiviral vector). The mice were injected intraperitoneally for 5 continuous days. Paw swelling was measured every 3 days with a slide gauge, and the degree of swelling was assessed by the increase in thickness compared with that of normal mice. Moreover, the arthritis scores of the CIA mice were calculated according to the following scoring criteria [21]: (0) no swelling or erythema, (1) local toe joint affected (swelling and erythema), (2) local toe joint and dorsum pedis affected, (3) entire paw affected (including ankle joint), and (4) entire paw affected (maximal erythema and swelling) with motor dysfunction.

### 2.7. Flow Cytometry

Splenic lymphocytes were harvested from the mice on day 42 after the first injection.

Subsets of lymph nodes T lymphocytes were stained with the following antibodies: CD3e (145-2C11), CD4 (GK1.5), INF-*γ* (XMG1.2), IL-17a (TC11-18H10), and FoxP3 (R16-715). Moreover, subsets of B lymphocytes from the spleen were analyzed by FACS with the following antibodies: CD19 (1D3) and IL-10 (JES5-16E3). Macrophages from the spleen were examined after being stained with CD11b (M1/70) and F4/80 (T45-2342) antibody. DCs from the spleen were examined after being stained with CD11c (HL3) antibody. All antibodies were purchased from BD Biosciences. All cells were acquired with a FACS C6 (BD Biosciences, USA) flow cytometer. The data were analyzed with FlowJo software.

### 2.8. ELISA

The levels of interferon gamma (IFN-*γ*), tumor necrosis factor alpha (TNF-*α*), interleukin-1 beta (IL-1*β*), interleukin 6 (IL-6), interleukin 17a (IL-17a), and interleukin 10 (IL-10) in mouse serum were measured by enzyme-linked immunosorbent assay (ELISA) kits (R&D Systems Minnesota, USA) according to the manufacturer's instructions. The concentrations of these cytokines were calculated from the standard curves.

### 2.9. Real-Time PCR

Real-time PCR was conducted on a Bio-Rad CFX Connect platform using the SYBR Fast qPCR Mix (Vazyme, China) to measure the expression of cytokines in joints. The mouse primers (forward primer, 5′GGGATCTAGAATGAGGATATTTGCTGGC; reverse primer, 5′GGGAGCGGCCGCCGTCTCCTCGAATTGTGT) were used in a polymerase chain reaction (PCR) to amplify PD-L1 cDNA and total inflammatory cytokine RNA from joint tissues as a template using the following thermal cycling conditions: first 50°C for 30 min and then denaturation at 94°C for 2 min, followed by 98°C for 10 s, with a final step at 68°C for 30 s. The amplifications were conducted for 25-30 cycles. The gene-specific primer sequences are shown in Supplementary Table 1.

### 2.10. Histological Assessment

Joint tissues were obtained after sacrifice. Joint tissues were fixed in formalin/PBS for 24 hours and embedded in paraffin after decalcification. Then, tissue sections were cut into sections 4 *μ*m thick and stained with hematoxylin and eosin (H&E). Histological scores of the joints were assessed by two independent observers. The histological score was assessed according to a previously described scoring system on the extent of synovitis, pannus formation, and bone and/or cartilage erosion, as the following criteria: 0: no signs of inflammation; 1: mild inflammation with hyperplasia of the synovial lining layer, minimal without cartilage erosion; 2 to 4: increasing degrees of inflammatory cell infiltrate or cartilage and bone erosion.

### 2.11. Statistical Analysis

GraphPad Prism 6 software (GraphPad, La Jolla, CA, USA) was used for statistical analysis. The data are shown as the mean ± SEMs, and *P* values were computed using a *t*-test or unpaired two-way analysis of variance (ANOVA). All reported differences were considered significant at *P* < 0.05.

## 3. Results

### 3.1. Construction and Characterization of PD-L1-Lentiviral Vector

**Figure 1:**
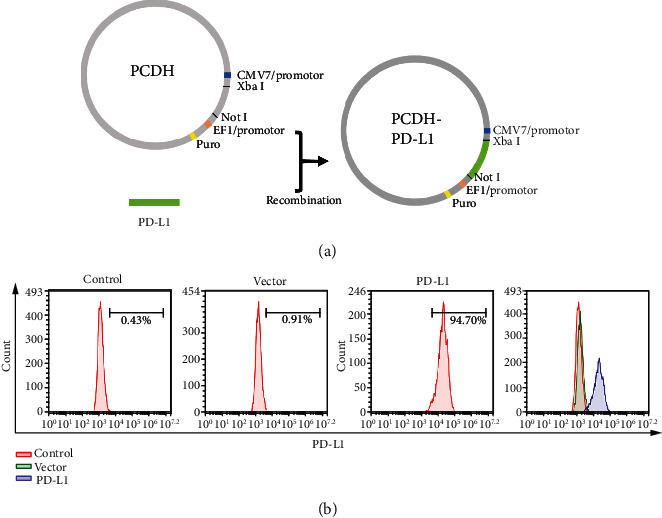
Generation and characterization of the PD-L1-lentiviral vector. (a) Schematic diagrams of the PD-L1-lentiviral vector containing PCDH cDNA and the therapeutic gene PD-L1. (b) FACS analysis of PD-L1 expression in MBMMSCs after transduction.

PD-L1 (873 bp) was successfully cloned into the PCDH vector (7384 bp), and the recombinant lentiviral vector was named PCDH-PD-L1. The promoter was CMV/7 promoter and enzyme cleavage sites were *XbaI* and *NotI* (Figure 1(a)). FACS analysis was conducted to confirm the expression of PD-L1 in transfected MBMMSCs, and data showed that more than 90% of the cells were able to successfully express PD-L1 (Figure 1(b)).

### 3.2. PD-L1-MBMMSCs Alleviated Disease Progression in the CIA Model

**Figure 2:**
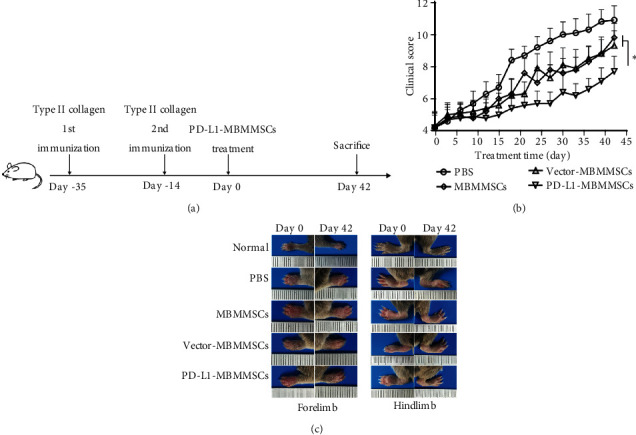
The effect of PD-L1-MBMMSCs on the development and pathological damage of CIA. (a) Schematic diagram of CIA model establishment and treatment. (b) Clinical scores of CIA mice after treatment with PBS, MBMMSCs, Vector-MBMMSC, and PD-L1-MBMMSCs. The data are shown as the means ± SEM (*n* = 10). ^∗^*P* < 0.05. (c) Joint appearance on day 42.

We induced the CIA model as described previously, and a schematic diagram of CIA model establishment and treatment is shown in Figure 2(a). Joint clinical score was recorded to elucidate the effects of PD-L1-MBMMSCs. Compared with MBMMSC and Vector-MBMMSC treatment, PD-L1-MBMMSC treatment significantly inhibited the development of CIA and ameliorated the clinical score (*P* < 0.05) (Figure 2(b)). The photographs clearly showed that paws of the mice treated with PD-L1-MBMMSCs were less swollen than the paws of other mice on day 42 (Figure 2(c)). In addition, paw swelling was measured by slide gauge during the experiment. Compared with that in the PBS group, the thickness of the forelimb and hindlimb in the PD-L1-MBMMSC treatment group was significantly reduced (*P* < 0.01) (Figure S2A-B). At day 42 after treatment, the joints were collected to obtain ankle sections, which were then stained with H&E. The results showed limited cartilage damage, synovial hyperplasia, inflammatory cell infiltration, and bone erosion in the PD-L1-MBMMSC treatment group after CIA induction (Figure S2C-D). These findings reveal that both MBMMSCs and PD-L1-MBMMSCs can suppress the development of CIA and alleviate disease severity and that PD-L1-MBMMSCs are more effective than MBMMSCs.

### 3.3. PD-L1-MBMMSCs Regulated Immune Cells in the Spleen

**Figure 3:**
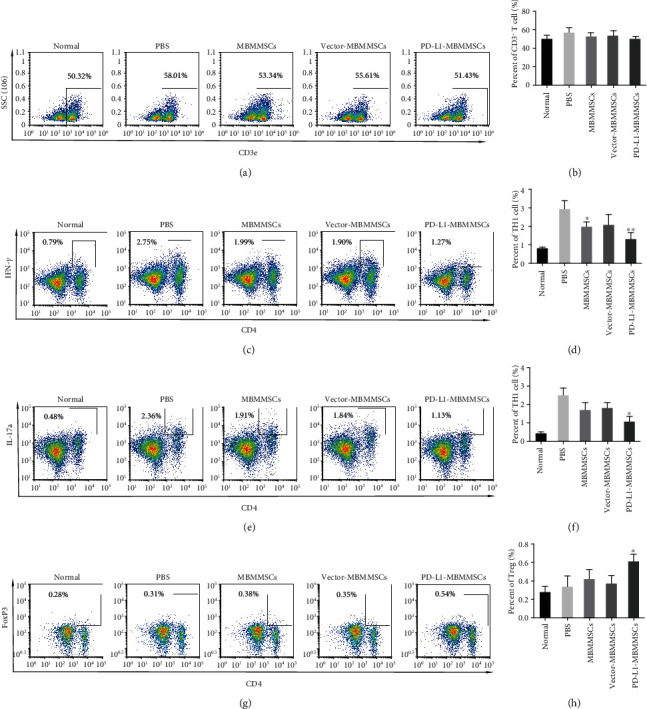
PD-L1-MBMMSCs induce changes in T cells. FACS analyses of (a, b) CD3^+^ T cells, (c, d) CD4^+^IFN-*γ*^+^ Th1 cells, (e, f) CD4^+^IL-17a^+^ Th17 cells, and (g, h) CD4^+^FOXP3^+^ Treg cells in the spleen of CIA mice (*n* = 3). The data are shown as the means ± SEM (*n* = 3). ^∗^*P* < 0.05 and ^∗∗^*P* < 0.01 versus PBS.

**Figure 4:**
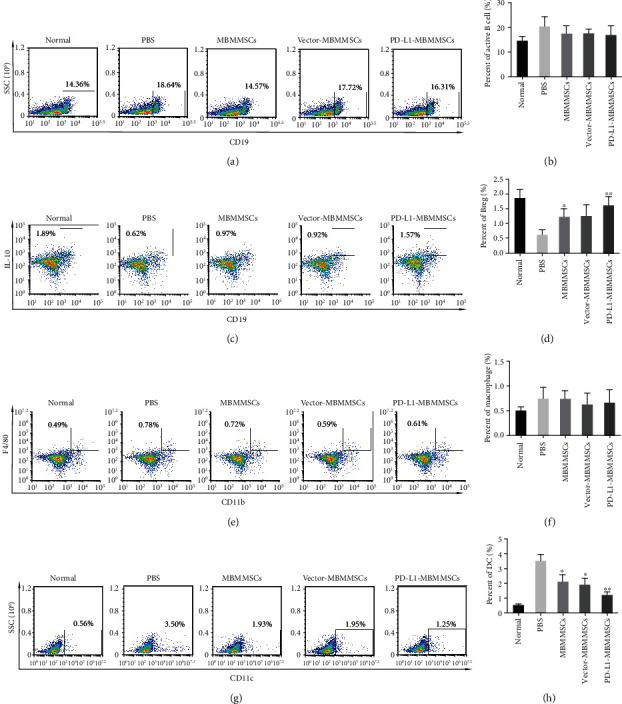
The effect of PD-L1-MBMMSCs on B cells, macrophages, and DCs. FACS analyses of (a, b) CD19^+^ B cells, (c, d) CD19^+^IL-10^+^ Breg cells, (e, f) CD11b^+^F4/80^+^ macrophages, and (g, h) CD11c^+^ DCs in the spleen of CIA mice (*n* = 3). The data are shown as the means ± SEMs (*n* = 3). ^∗^*P* < 0.05 and ^∗∗^*P* < 0.01 versus PBS.

Immune cells play vital roles in the pathogenesis of arthritis. To investigate the effect of PD-L1-MBMMSCs on immune cells in the spleen, T cells, B cells, macrophages, and dendritic cells (DCs) in the spleen of CIA mice were analyzed by FACS. First, we measured total T cells, and there were no significant differences in the numbers of CD3^+^ T cells between the groups (Figures 3(a) and 3(b)). CD4^+^ T cells are essential for CIA induction and development. FACS analysis showed that the numbers of CD4^+^IFN-*γ*^+^ Th1 cells were decreased in the MBMMSC and PD-L1-MBMMSC groups (Figures 3(c) and 3(d)). Furthermore, CD4^+^IL-17a^+^ Th17 cells were reduced after PD-L1-MBMMSC treatment (Figures 3(e) and 3(f)). Regulatory T (Treg) cells are a class of T cells with immunomodulatory effects. In our study, the numbers of splenic CD4^+^FoxP3^+^ Treg cells in the PD-L1-MBMMSC-treated group were significantly higher than those in the other groups (Figures 3(g) and 3(h)). In addition to T cells, we also examined other lymphocytes, including B cells, macrophages, and DCs. CIA mice showed no significant decreases in active CD19^+^ B cells (Figures 4(a) and 4(b)). The number of CD19^+^IL10^+^ regulatory B (Breg) cells in the spleen was similar to that of splenic Treg cells. Compared with PBS-treated mice, MBMMSC-treated mice and PD-L1-MBMMSC-treated mice showed dramatic recovery of the number of Breg cells (Figures 4(c) and 4(d)). No changes in macrophages were observed (Figures 4(e) and 4(f)), whereas PD-L1-MBMMSCs markedly decreased the number of CD11c^+^ DCs (Figures 4(g) and 4(h)). These results suggest that PD-L1-MBMMSCs inhibit CIA development by regulating the numbers of T cells, B cells, and DCs in the spleen.

### 3.4. PD-L1-MBMMSCs Regulated Inflammatory Cytokine Production

**Figure 5:**
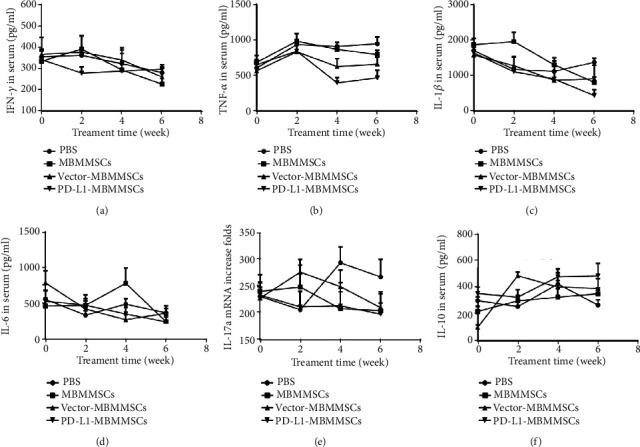
PD-L1-MBMMSCs alter the secretion of cytokines in the serum. ELISA measurement of (a) IFN-*γ*, (b) TNF-*α*, (c) IL-1*β*, (d) IL-6, (e) IL-17a, and (f) IL-10 concentrations in CIA mouse serum. Serum was obtained once every 2 weeks beginning on the first day of PD-L1-MBMMSC injection. The data are shown as the means ± SEM (*n* = 3).

**Figure 6:**
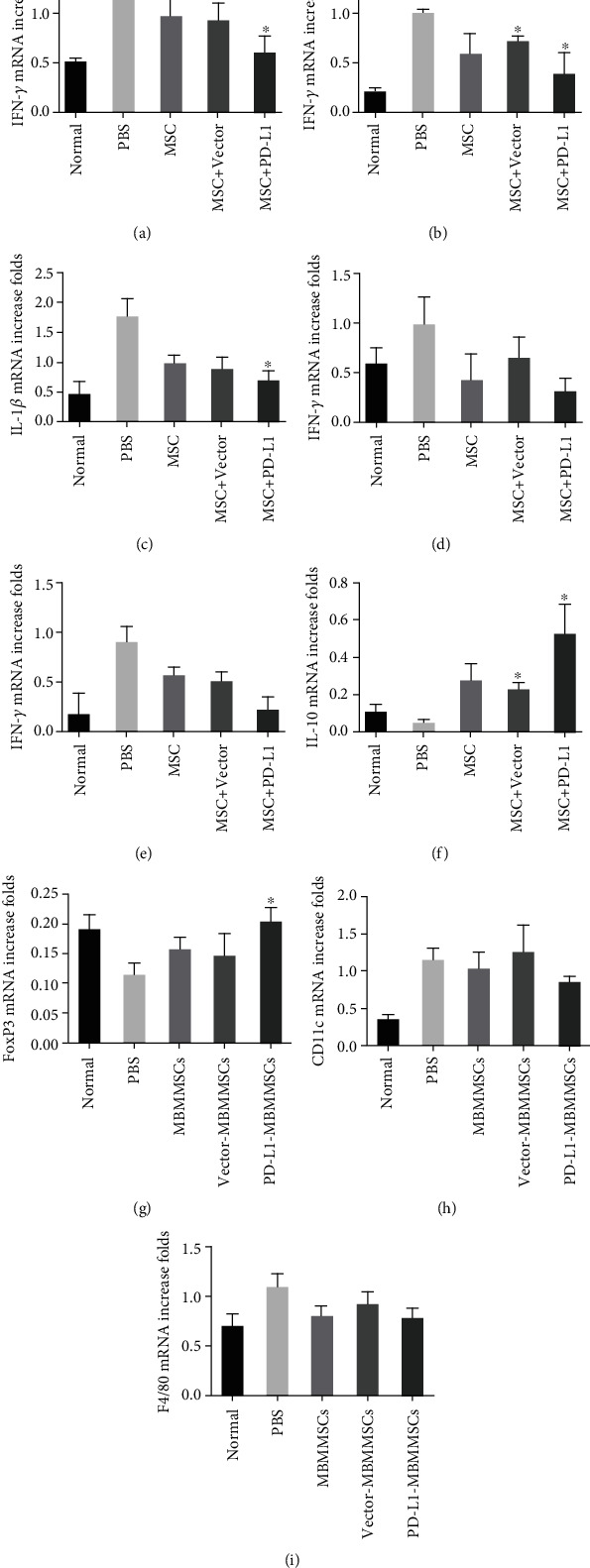
PD-L1-MBMMSCs regulate the expression of cytokines in the joint. Real-time PCR analysis of (a) IFN-*γ*, (b) TNF-*α*, (c) IL-1*β*, (d) IL-6, (e) IL-17a, and (f) IL-10 mRNA levels in joints from CIA mice on day 42. The data are shown as the means ± SEM (*n* = 3). ^∗^*P* < 0.05 and ^∗∗^*P* < 0.01 versus PBS.

Lymphocytes can secrete various cytokines, which have also been showed to have important roles in CIA pathogenesis. To assess whether PD-L1-MBMMSCs affected lymphocyte production of inflammatory cytokines, we examined serum expression levels of IFN-*γ*, TNF-*α*, IL-1*β*, IL-6, IL-17a, and IL-10 each week. Proinflammatory factors (IFN-*γ*, TNF-*α*, IL-1*β*, IL-6, and IL-17a) were gradually reduced in the sera of mice treated with PD-L1-MBMMSCs compared with those of control-treated mice, and the levels of proinflammatory factors in the PD-L1-MBMMSC group were lower than those in the other groups (Figures 5(a)–5(e)). In contrast, the anti-inflammatory cytokine IL-10 was increased after PD-L1-MBMMSC treatment (Figure 5(f)). Cytokine levels were also measured in joint tissues by real-time PCR. Consistent with the serum analysis results, proinflammatory cytokine gene expression in joints tissues was downregulated and anti-inflammatory cytokine gene expression was upregulated on day 42 in the PD-L1-MBMMSC group (Figures 6(a)–6(i)). These results indicate that PD-L1-MBMMSCs regulate inflammatory cytokines production in serum and joints.

## 4. Discussion

RA is a chronic autoimmune and systemic disease that mainly affects the joints. Long-term treatment results in approximately 40% of RA patients becoming resistant and not responding to any available clinical treatments. Therefore, novel and effective treatments for RA are urgently needed [22]. Stem cell therapy is emerging as a potential treatment for RA. MSC-based therapy is a promising new method for the treatment of arthritis that has been further developed in recent years [23, 24]. In this study, we modified MSCs with PD-L1 and demonstrated that PD-L1-expressing MSCs inhibited the development of arthritis in mice more effectively than unmodified MSCs.

MSCs isolated from the bone marrow, umbilical cord, or adipose tissue are pluripotent progenitors that can differentiate into cells that can form tissues, such as bone and cartilage. Studies have shown that mesenchymal stem cells can differentiate into osteoblasts during isolation and culture in vitro [25]. Moreover, MSCs exhibit immunosuppressive activity due to paracrine actions and interactions with different immune cells, in addition to their multilineage differentiation potential [26]. Our study showed that MBMMSCs could regulate immune cells in CIA mice, including T cells, B cells, and DCs. However, the regulatory effect of MBMMSCs was mainly associated in Th1 cells, Breg cells, and DCs. Additionally, PD-L1-MBMMSCs could affect Th17 cells and Treg cells at the same time. The levels of cytokines in serum and joint tissue were also assessed. MBMMSCs downregulated the levels of IFN-*γ*, TNF-*α*, IL-1*β*, IL-6, and IL-17a and upregulated the expression of IL-10. However, PD-L1-MBMMSCs exhibited a more significant regulatory effect on these cytokines than MBMMSCs, suggesting that the anti-inflammatory effect of MBMMSCs on CIA mice was enhanced after PD-L1 transfection.

Most autoimmune diseases are caused by dysfunction in the complex immune tolerance system. PD-1 is one aspect of this system [27]. It has been reported that PD-1 inhibits T cell proliferation and that PD-L1 and/or PD-L2 expressed by a variety of malignant tumor cells can mediate escape from host immunity by regulating T cells [28, 29]. A previous study confirmed that the synovium in RA can express PD-L1, PD-L2, B7-H3, and B7-H4 [30]. PD-L1 Fc administration increased PD-1 activity and inhibited T cell proliferation, leading to a reduction in arthritis. Consistent with previous research, our study proved that PD-L1 could decrease the numbers of Th1 and Th7 cells and upregulate the number of Treg in CIA mice. In addition to T cells, PD-1/PD-L1 also affects B cells and monocytes [31]. PD-L1-MBMMSC treatment resulted in a decrease in activated B cells and the upregulation of Breg cells in this study. DCs were also suppressed by PD-L1 in CIA mice. Moreover, PD-L1 could significantly regulate the expression of cytokines in the serum and joints of CIA mice. Therefore, the PD-L1 pathway is a promising therapeutic target in RA.

In summary, we demonstrated that the administration of genetically modified MBMMSCs overexpressing PD-L1 improves the severity of experimental arthritis by not only suppressing autoimmune response to lymphocytes but also regulating cytokine production. Although MBMMSCs alone showed anti-inflammatory activity, their effects were weaker than those of PD-L1-MBMMSCs. Moreover, the antiarthritis effects on mice treated with PD-L1-MBMMSCs seem to be caused by the cumulative effects of the MBMMSCs themselves and PD-L1 secretion. These data provide new insights into the advantages of MBMMSCs as anti-inflammatory cells in RA therapy that can suppress the autoimmune response and deliver desirable genes, such as PD-L1, which is an effective strategy for RA treatment.

## Data Availability

The initial data used to support the findings of this study are available from the corresponding author upon request.

## Abbreviations

PD-L1: Programmed cell death 1 ligand
PD-1: Programmed cell death 1 receptor
RA: Rheumatoid arthritis
MBMMSCs: Mouse bone marrow mesenchymal stem cells
CIA: Collagen-induced arthritis
PD-L1-MBMMSCs: PD-L1-transfected MBMMSCs
TNF: Tumor necrosis factor
IL: Interleukin
ITSM: Tyrosine-based switch motif
Syk: Spleen tyrosine kinase
PI3K: Phosphatidylinositol 3-kinase
MSCs: Mesenchymal stem cells
FBS: Fetal bovine serum
MOI: Multiplicity of infection
IFN-*γ*: Interferon gamma
TNF-*α*: Tumor necrosis factor alpha
IL-1*β*: Interleukin-1 beta
IL-6: Interleukin 6
IL-17a: Interleukin 17a
IL-10: Interleukin 10
ELISA: Enzyme-linked immunosorbent assay
PCR: Polymerase chain reaction
H&E: Hematoxylin and eosin
ANOVA: Unpaired two-way analysis of variance
DCs: Dendritic cells
Treg: Regulatory T
Breg: Regulatory B.

## Supplementary Materials

Supplementary Materials Figure S1: the detection of the features of MSCs. FACS analysis of CD105^+^, CD73^+^, CD90^+^, CD34^−^, and CD11b^−^. Figure S2: the state of the damage to the joint. (A, B) Forelimb and hindlimb paw thickness in the different mouse groups. The data are shown as the means ± SEM (*n* = 10). ^∗∗^*P* < 0.01. (C) H&E staining of knee joint. The magnification is ×40. The scale bar is 200 *μ*m. (D) Pathological scores of the knee joint. The data are shown as the means ± SEM (*n* = 3). ^∗^*P* < 0.05 and ^∗∗^*P* < 0.01 versus PBS. Supplementary Table 1: primer sequences for real-time PCR.
