# Supplementary material for: Programmed Cell Death Ligand 1-Transfected Mouse Bone Marrow Mesenchymal Stem Cells as Targeted Therapy for Rheumatoid Arthritis
Source: Biomed Res Int. 2021 Aug 30;2021:5574282. doi: 10.1155/2021/5574282 (PMC8421163; doi:10.1155/2021/5574282)
Supplement: Supplementary Materials — Figure S1: the detection of the features of MSCs. FACS analysis of CD105+, CD73+, CD90+, CD34−, and CD11b−. Figure S2: the state of the damage to the joint. (A, B) Forelimb and hindlimb paw thickness in the different mouse groups. The data are shown as the means ± SEM (n = 10). ∗∗P < 0.01. (C) H&E staining of knee joint. The magnification is ×40. The scale bar is 200 μm. (D) Pathological scores of the knee joint. The data are shown as the means ± SEM (n = 3). ∗P < 0.05 and ∗∗P < 0.01 versus PBS. Supplementary Table 1: primer sequences for real-time PCR. [file 5574282.f1.doc]

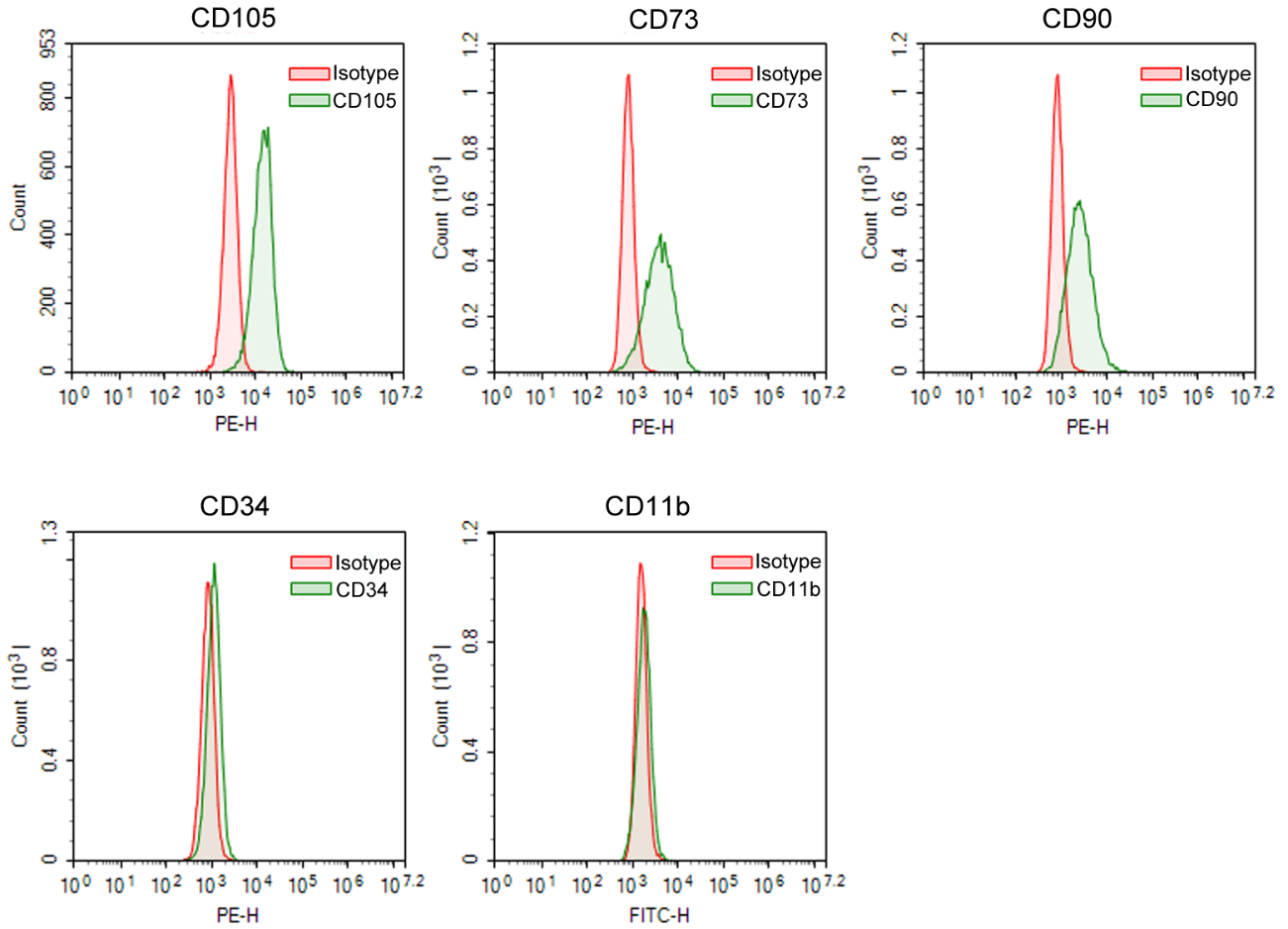


**Fig. S1. The detection of the features of MSCs.** FACS analysis of CD105+, CD73+, CD90+, CD34- and CD11b-.


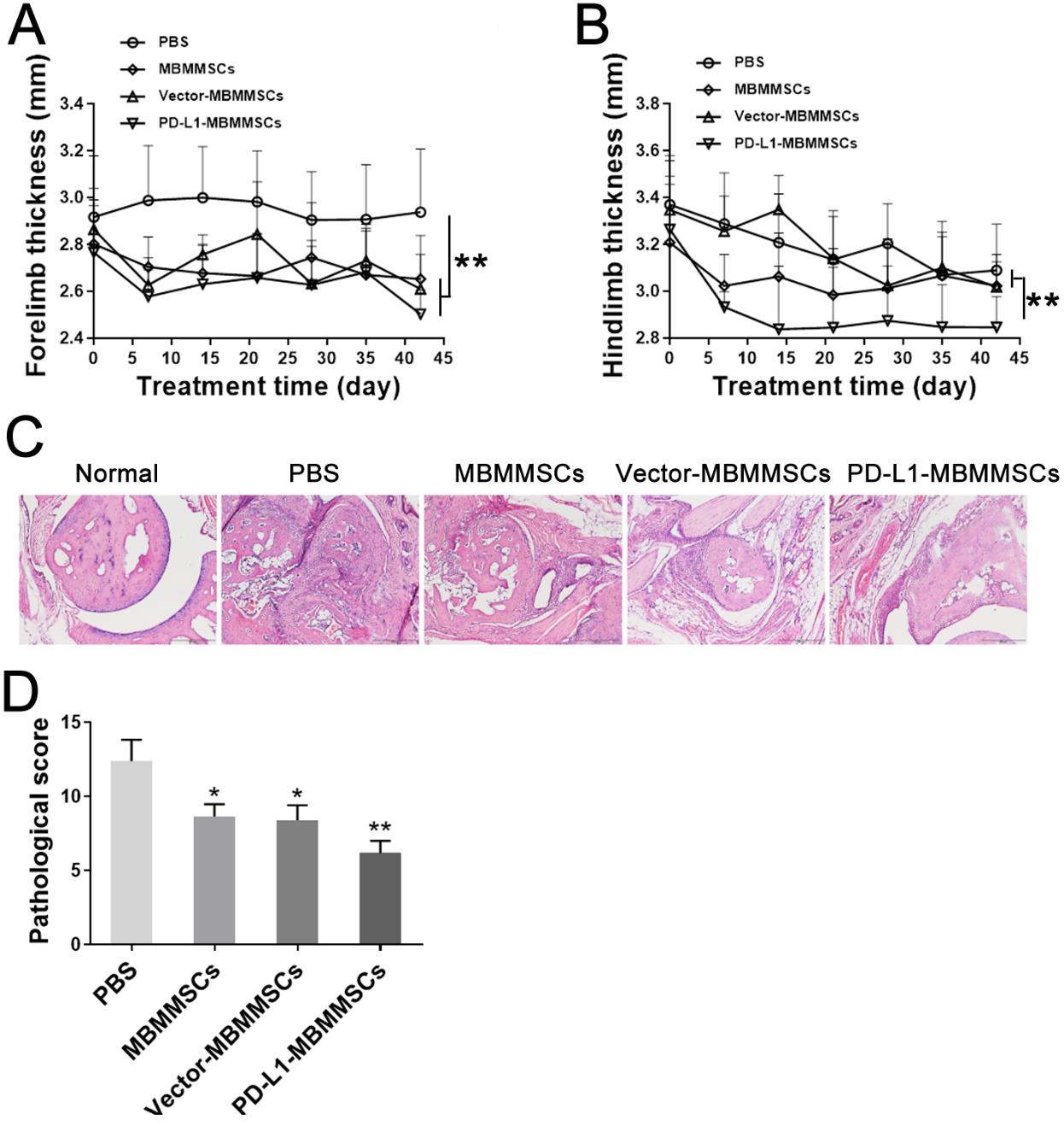


**Fig. S2. The state of the damage to the joint.** (A-B) Forelimb and hindlimb paws thickness in the different mouse groups. The data are shown as the means ± SEM (n=10). ***P* < 0.01. (C) H&E staining of knee joint. The magnification is 40×. The scale bar is 200 μm. (D) Pathological scores of the knee joint. The data are shown as the means ± SEM (n=3). **P* < 0.05, ***P* < 0.01 versus PBS.
